# Exposure to Neighborhood Green Space and Mental Health: Evidence from the Survey of the Health of Wisconsin 

**DOI:** 10.3390/ijerph110303453

**Published:** 2014-03-21

**Authors:** Kirsten M. M. Beyer, Andrea Kaltenbach, Aniko Szabo, Sandra Bogar, F. Javier Nieto, Kristen M. Malecki

**Affiliations:** 1 Division of Epidemiology, Institute for Health and Society, Medical College of Wisconsin, 8701 Watertown Plank Rd., Milwaukee, WI 53226, USA; E-Mail: drea2340@gmail.com; 2 Division of Biostatistics, Institute for Health and Society, Medical College of Wisconsin, 8701 Watertown Plank Rd., Milwaukee, WI 53226, USA; E-Mail: aszabo@mcw.edu; 3 PhD Program in Public and Community Health, Institute for Health and Society, Medical College of Wisconsin, 8701 Watertown Plank Rd., Milwaukee, WI 53226, USA; E-Mail: sbogar@mcw.edu; 4 Department of Population Health Sciences, University of Wisconsin-Madison, Wisconsin Alumni Research Foundation, 610 Walnut St., Madison, WI 53726, USA; E-Mails: fjnieto@wisc.edu (F.J.N.); kmalecki@wisc.edu (K.M.M.)

**Keywords:** green space, nature, neighborhood environment, mental health, population-based surveys, United States

## Abstract

Green space is now widely viewed as a health-promoting characteristic of residential environments, and has been linked to mental health benefits such as recovery from mental fatigue and reduced stress, particularly through experimental work in environmental psychology. Few population level studies have examined the relationships between green space and mental health. Further, few studies have considered the role of green space in non-urban settings. This study contributes a population-level perspective from the United States to examine the relationship between environmental green space and mental health outcomes in a study area that includes a spectrum of urban to rural environments. Multivariate survey regression analyses examine the association between green space and mental health using the unique, population-based Survey of the Health of Wisconsin database. Analyses were adjusted for length of residence in the neighborhood to reduce the impact of neighborhood selection bias. Higher levels of neighborhood green space were associated with significantly lower levels of symptomology for depression, anxiety and stress, after controlling for a wide range of confounding factors. Results suggest that “greening” could be a potential population mental health improvement strategy in the United States.

## 1. Introduction

The presence of green space, such as forests and parks, is now widely viewed as a health-promoting characteristic of residential environments, and has been linked to benefits such as recovery from mental fatigue [1,2,3,4,5,6,7], stress reduction [8,9,10] neighborhood social cohesion [11], reductions in crime, violence and aggression [12,13,14,15], reduced morbidity in multiple disease categories [16,17,18] and better self-reported health [17,18,19].

Some studies have demonstrated that the relationship between green space and health is stronger among lower socioeconomic status groups, the elderly, and others who stay home during the day, providing some support for the notion that the size of the effect of local green space on health is related to the amount of an individual’s exposure to the local environment [17,19]. These findings suggest that increasing neighborhood access to green space could be a cost-effective strategy to improving health and reducing health disparities, as lower socioeconomic status groups have a more limited ability to travel beyond local neighborhoods, resulting in increased dependence on local environments for healthy lifestyles and exposures [7,17,19,20,21,22,23]. Given evidence suggesting health benefits of exposure to green space and stronger effects among some racial, ethnic, or socioeconomic groups, it has been suggested that green spaces could be “systematically deployed to mitigate health inequalities” in addition to improving health overall [7,23].

However, while green space holds great promise as an innovative, place-based solution to improving population health, understanding where and for whom green space confers health benefits has proven complex. Some evidence and theoretical guidance has suggested that several main pathways may be important in linking green space exposure to health benefits. Green space can have direct protective effects against health hazards posed by air pollution, extreme temperature, and noise pollution [24,25]; has been associated with increased health promoting behaviors such as physical activity [23,26]; and linked to increased levels of social support, social cohesion, and sense of community [11,27,28,29]; and to mental health benefits such as stress reduction [8,10,23]; buffering between stressors and health outcomes [30]; and attention restoration that reduces mental fatigue [1,2,3,4,5,6]. Recent research has linked green space directly to biomarkers of stress and attention—diurnal variation of salivary cortisol [8,10] and brain waves as measured by portable EEG devices [9,31]—suggesting a biologically plausible link between exposure to green space and reduction of stress and mental fatigue.

The mental health benefits conferred by green space are of particular interest given a growing body of knowledge that emphasizes stress responses as a main link between neighborhood conditions and health outcomes. Attention Restoration Theory posits that experiences in natural environments can reduce mental fatigue and restore the capability for directed attention. Directed attention is employed “when something (does) not of itself attract attention, but when it (is) important to attend nonetheless” [2]. Maintaining this focus requires mental effort, which can lead to mental fatigue. In order to recover from mental fatigue, an individual must have the opportunity to relax directed attention. One way to accomplish this is to engage in another kind of attention—fascination attention—which occurs involuntarily and does not require the same mental effort as directed attention. Scholars argue that natural environments have the inherent capacity to fascinate, thereby providing a restorative experience that enables recovery from mental fatigue. This may be particularly relevant when considering the directed attention demands of fast-paced, modern, urban environments [2]. 

Numerous studies, including a large body of experimental work in the field of environmental psychology, have empirically linked nature exposure with attention restoration and stress reduction [1,2,3,4,5,6], as well as the buffering between stressful life events and psychological outcomes [32]. Early work focused on visual images of nature scenes and resultant impacts on preferences for different landscapes and measures such as emotions and heart rate [33,34]. More recent work has emphasized experimental designs incorporating walks/runs in natural areas and additional measurement techniques, including the Digit Span Backwards (DSB) test and other tests of cognitive function. Several studies from the Landscape and Human Health laboratory at the University of Illinois at Urbana-Champaign have provided strong evidence of nature’s benefits for children affected by Attention Deficit Hyperactivity Disorder (ADHD) [1,22,35,36,37]. In particular, one study engaged children with ADHD in walks in several different environments, finding improvements in attention after walking in a park, as compared to walks in a downtown and neighborhood setting; the study found significant effect sizes comparable to those reported for common pharmaceutical therapies for ADHD [1]. Several recent reviews provide good coverage of the current evidence base and may be consulted for further detail [38,39,40]. In particular, a meta-analysis by Bowler *et al.* (2010) identified effect sizes and significant levels indicative of improvement in energy, anxiety, anger, fatigue and sadness with exposure to natural environments [38], with less evidence of an impact on attention, tranquility, blood pressure or cortisol. Findings of this 2010 review can be contrasted with those from the studies of children with ADHD described above, as well as with more recent work that has linked green space with biomarkers of stress (cortisol) [8,10] and with changes in brain waves indicative of a more “meditative” cognitive state [9,31]. 

Previous work has suggested that greenspace may be linked to lower levels of depression, anxiety and/or stress, and that more research is needed [41,42]. More evidence is needed to inform the development and implementation of effective neighborhood-level interventions that harness the salutogenic properties of green space to improve health and reduce disparities in different geographical settings. While population level studies have examined the role of green space, stress and mental health in several European countries [8,10,16,43] and New Zealand [44], and several US studies have examined the health benefits of green space more generally [45,46,47,48], less is known about green space and mental health relationships at the population level in settings across the United States (US). In the US, a recent study demonstrated that a program to green vacant lots in Philadelphia significantly reduced stress [12] and another found a weak association between green space and mental health in Miami [41]. A third study found a positive impact of green space for stress reduction in Chicago, with park spaces demonstrating a larger effect size than the vegetation level overall [49]. A recent review concluded that much of the evidence of a psychological benefit of green space is from qualitative data and non-peer reviewed sources, and argued that more research is needed to quantify the association between green spaces and health [39]. 

Given an evidence base suggesting associations between environmental green space and both physical and mental health outcomes in non-US settings, the potential for green space to contribute to reductions in health inequities, and the significant and persistent patterns of place-based health inequities associated with the history of residential racial segregation in the United States, there is a great need for more research examining the potential role for green space as a population health intervention in the US. Further, few studies have considered the role of green space in non-urban settings. This study contributes a population-level perspective from the United States to examine the relationship between environmental green space and mental health outcomes in a study area that includes a spectrum of urban to rural environments. In addition, in recognition of previous work that has found variation in impact of different types of green spaces [49] and a body of work that emphasizes trees as a special and important category of green space that deserves particular attention [20,45,46,50,51,52], we examine several measures of green space in the present study.

## 2. Methods

### 2.1. Data

This is a cross-sectional study of the relationship between neighborhood green space and mental health among a probability sample of Wisconsin residents. We analyzed data from the Survey of the Health of Wisconsin (SHOW) database. SHOW is an ongoing survey, established in 2008, which is modeled after the National Health and Nutrition Examination Survey (NHANES). It includes information collected through interviews, physical exams, and biospecimens from a representative sample of Wisconsin residents. Records are geocoded by street address to allow linkage with physical and social environmental characteristics. Details of SHOW methods and rationale have been published previously [53]. Briefly, each year a representative sample of civilian, non-institutionalized adult (age 21–74 years) residents of the State of Wisconsin is selected from random households using a two-stage probability-based cluster sampling approach, stratified by region and poverty level. Since program initiation in 2008, recruitment goals have ranged from 400 to 1,000 participants per year. Participation rates have improved over time, reaching 63% in 2011. We analyzed data from the 2008–2009, 2010 and 2011 cohorts of SHOW, for a total sample of 2,479 individuals nested in 229 Wisconsin Census Block Groups (primary sampling units).

### 2.2. Outcome Measures

Our outcome measures comprise the three scales of the 42-item Depression Anxiety and Stress Scales (DASS) instrument [54], indicating symptomology for depression (self-disparaging; dispirited, gloomy, blue; convinced that life has no meaning or value; pessimistic about the future; unable to experience enjoyment or satisfaction; unable to become interested or involved), anxiety (apprehensive, panicky; trembly, shaky; aware of dryness of the mouth, breathing difficulties, pounding of the heart, sweatiness of the palms; worried about performance and possible loss of control) and stress (over-aroused, tense; unable to relax; touchy, easily upset; irritable; easily startled; nervy, jumpy, fidgety; intolerant of interruption or delay). Higher scores on these measures indicate poorer mental health states. We analyze these scores as continuous measures of symptomology in the present analyses. To guide interpretation, it is beneficial to know that each of the 3 DASS scales can be categorized into 5 ordinal categories (Normal, Mild, Moderate, Severe, Extremely Severe) using the following cut-off points, respectively: 10, 14, 21, 28 for depression; 8, 10, 15, 20 for anxiety; and 15, 19, 26, 34 for stress [54]. 

### 2.3. Individual-level Confounders

We controlled for a range of individual level characteristics that may affect the relationship between green space and health [14,23,26] including age (<35, 35–44, 45–54, 55–64, 65–74 years), gender (female, male), race/ethnicity (White, Non-White), and marital status (Never married, Married/Living with a partner, Separated/Divorced/Widowed). We controlled for individual level socio-economic status with measures of education (less than high school, high school degree, some college/associate’s degree, bachelor’s degree, above bachelors or professional degree), annual household income (<$20,000, $20,000–34,999, $35,000–49,999, $50,000–74,999, ≥$75,000), occupational status (working at a job or business, with a job or business but not at work—vacation or sick leave, not working but looking for work, not working at a job or business and not looking for work), residence type (owned, rented, other), and type of health insurance (uninsured, private only, public/government, multiple carriers, other). 

### 2.4. Neighborhood Definition

The US census block group is a geographical unit smaller than the census tract, containing approximately 600 to 3,000 people [55], and is the primary sampling unit for SHOW data collection. Because the time period of this study extends across the year 2010, at which point US Census boundaries were redefined, it is difficult to align socio-demographic information from the US Census spatially and temporally with data from SHOW at the block group level. Additionally, margins of error for some measures at small geographies can be a cause of concern. Block groups are particularly affected by the change in boundaries, as they are comprised of the smallest Census geographic units—census blocks—for which socio-demographic information is unavailable, and thus cannot be aggregated. Because of these concerns, we elected to use socio-demographic data associated with the larger unit that encompasses the block group—the US Census tract.

### 2.5. Neighborhood-level Control Measures

To measure socio-demographic characteristics of neighborhoods, we used tract level information for years closely aligned with our survey data, from the American Community Survey for the years 2007–2011, using Tiger/Line 2011 census tract boundaries. Consistent with previous research [19], we controlled for the level of urbanicity using both Rural and Urban Commuting Area (RUCA) codes for 2003 (the most recent available) and population density (Census 2010) at the tract level. In addition, we incorporated measures of socioeconomic status (median household income, percent below poverty level), residential instability (percent in same house 1 year ago), percent owner occupied households, and percent unemployment. Because of the legacy of racial segregation and close linkages among segregation and socioeconomic status in the US [56], we also incorporated a measure of residential racial segregation (percent African American).

### 2.6. Level of “Greenness”: NDVI

**Figure 1:**
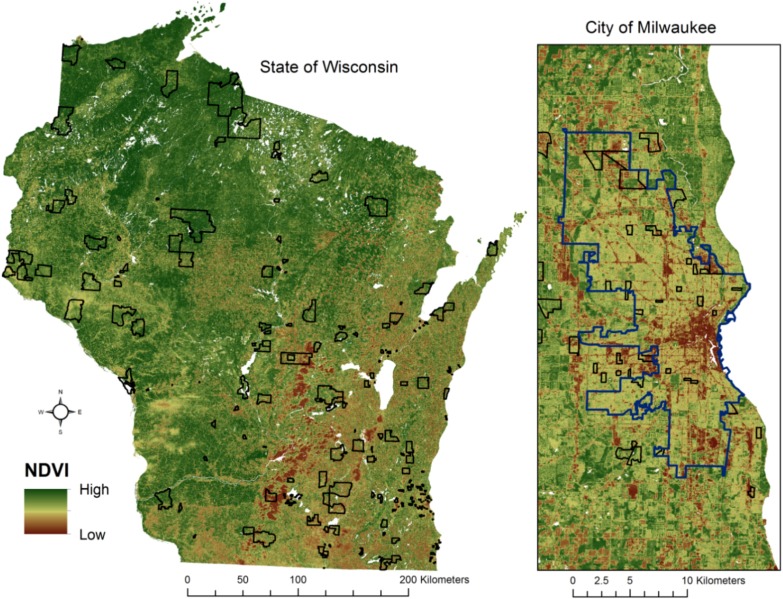
Normalized Difference Vegetation Index and SHOW Block Groups, Wisconsin, 2009.

Neighborhood greenness was operationalized using the normalized difference vegetation index (NDVI), which has been shown to be effective in capturing neighborhood greenness for epidemiologic purposes [57] and is a commonly used index of greenness because of its “simplicity, good sensitivity in vegetation changes and best dynamic range” [58]. NDVI was calculated per pixel in Erdas IMAGINE software using Landsat 5 satellite imagery for July 2009 at 30 m resolution, and the mean NDVI value was calculated for each SHOW Census Block Group using zonal statistics in Esri ArcGIS software [59]. Block group boundaries containing significant cloud cover were cropped to eliminate cloud-covered areas and water bodies were removed before calculating average NDVI values for each CBG. The results of the NDVI calculation and location of the SHOW census block groups that comprise the study area is shown in Figure 1. Higher values of the NDVI correspond to higher density of healthy vegetation. NDVI values can be loosely interpreted as follows: 0.1 or less = barren rock, sand or snow, 0.2–0.5 = sparse vegetation (shrubs, grassland), 0.6–0.9 = dense vegetation such as temperate and tropical rainforests and crops at peak growth stage) [60].

### 2.7. Percent Tree Canopy Coverage

**Figure 2:**
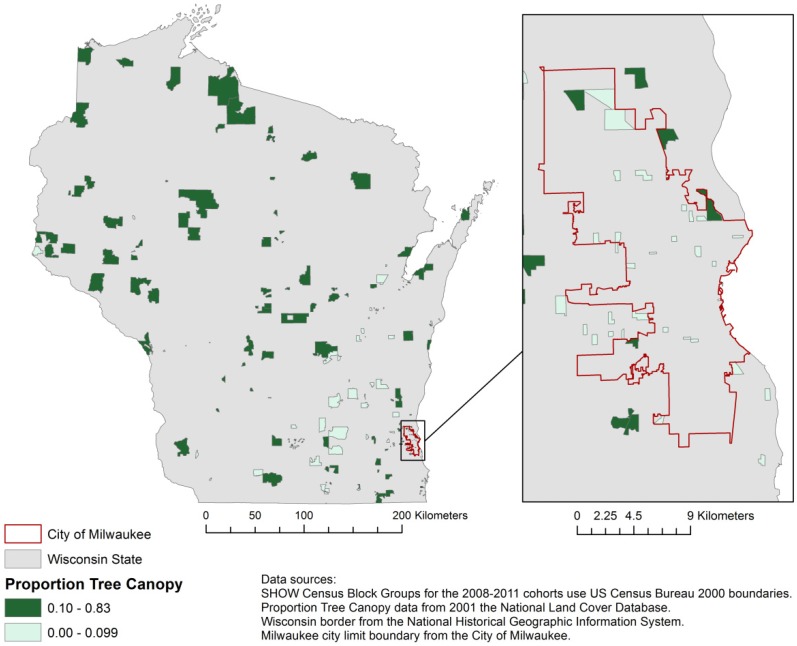
2001 Proportion Tree Canopy and SHOW Block Groups.

Tree canopy coverage was measured using 2001 Percent Tree Canopy data from the National Land Cover Database [61]. This is the most recent data readily available on Wisconsin tree canopy and is helpful in determining the independent influence of trees and the forested environments they represent, as opposed to greenness that may originate from agriculture or other land uses. The median percent tree canopy in SHOW census block groups is 10%; Figure 2 compares census block groups with 10% or more tree canopy to block groups with less than 10% canopy.

### 2.8. Statistical Analysis

All statistical analyses were undertaken in Stata SE/12 [62] and accounted for the SHOW survey design and population weights. Descriptive statistics were adjusted for the SHOW survey design and sampling weights (tabout command with svy option in STATA). Bivariate analyses compared all variables of interest between areas with less than 10% tree canopy or 10% or more tree canopy, as the median proportion tree canopy in the study area was 10%. Regression analyses were completed using survey data analysis commands available in Stata SE/12 (svy linearized: regress). Because our main green space predictors—NDVI and percent tree canopy—are highly correlated (*r* = 0.83), we modeled each of the three outcomes using three different continuous measures of neighborhood greenness—NDVI, percent tree canopy, and average of NDVI and percent tree canopy. Multivariate linear regression models were adjusted for all individual level (age, gender, race and ethnicity, education, income, marital status, insurance status) and neighborhood level (urbanicity/rurality, population density, education, instability, unemployment, poverty, housing tenure, percent African American, median household income) factors. In addition, models were adjusted for length of residence of the respondent in the neighborhood environment measured. Confounders were incorporated as dummy variables in regression analyses. Multicollinearity was assessed using a criterion of variance inflation factor >10, but no problems were detected.

## 3. Results and Discussion

### 3.1. Sample Characteristics and Bivariate Analyses

Sample characteristics and unadjusted, bivariate analyses are displayed in Table 1. Shorter length of residence was more common in neighborhoods with less than 10% tree canopy. Higher proportions of older age groups—particularly age group 55–64 years—lived in environments with more than 10% tree canopy, and higher proportions of younger age groups—particularly those under 44 years of age—lived in environments with less than 10% tree canopy. A higher proportion of individuals living in environments with less than 10% tree canopy were in the lowest income group, making less than $20,000 per year. Marital status differed significantly, with higher proportions of individuals who had never married or were divorced/separated/widowed living in areas with less than 10% tree canopy. 

**Table 1:** Sample Characteristics by Percent Tree Canopy.

		Less than 10%	10% or More	Total %	*p*-value
**Length of Residence (in years)**					**Pr = 0.005**
	Less than 1 year	13.9	7.7	10.9	
	1–2 Years	15.5	11.5	13.6	
	More than 2 but Fewer than 5 Years	17.5	14.4	16.0	
	More than 5 but Fewer than 10 Years	16.0	17.0	16.5	
	More than 10 but Fewer than 20 Years	13.8	17.2	15.4	
	More than 20 but Fewer than 40 Years	10.6	12.3	11.4	
	More than 40 Years	12.7	19.8	16.1	
**Age**					**Pr = 0.004**
	<35	29.7	20.4	25.3	
	35–44	23.1	18.5	20.9	
	45–54	22.7	28.9	25.7	
	55–64	15.2	19.8	17.4	
	65–74	9.3	12.3	10.7	
**Gender**					**Pr = 0.291**
	Male	49.3	51.2	50.2	
	Female	50.7	48.8	49.8	
**Race and Ethnicity**					**Pr = 0.172**
	Non-Hispanic White	84.4	88.8	86.5	
	Non-white	15.6	11.2	13.5	
**Educational Attainment**					**Pr = 0.068**
	Less than High School	9.1	5.6	7.4	
	High School degree/GED	18.3	23.1	20.6	
	Some post-secondary	39.1	38.4	38.8	
	Bachelor’s degree	23.9	21.1	22.6	
	Post-graduate work	9.6	9.6	10.6	
	Missing	0.0	0.2	0.1	
**Household Income**					**Pr = 0.007**
	<$20,000	18.3	9.0	13.8	
	$20,000–34,999	15.2	15.8	15.5	
	$35,000–49,999	13.1	15.5	14.3	
	$50,000–74,999	20.7	22.5	21.5	
	$75,000+	28.1	34.4	31.1	
	Missing	4.6	2.9	3.8	
**Marital Status**					**Pr = 0.000**
	Never married	22.8	10.7	17	
	Married or living with partner	61.1	77.2	68.8	
	Divorced, Separated or widowed	16.1	11.7	14	
	Missing	0.0	0.4	0.2	
**Occupational Status**					**Pr = 0.195**
	Working	63.4	69.1	66.1	
	With a job or business, but not at work	5.1	4.3	4.7	
	Not working but looking for work	8.7	6.3	7.6	
	Not working and not looking for work	22.7	20.1	21.5	
	Missing	0.1	0.3	0.2	
**Insurance Type**					**Pr = 0.025**
	Uninsured	7.5	7.2	7.4	
	Private	61.9	66.8	64.2	
	Public	13.3	7.4	10.5	
	Multiple	16.0	16.6	16.3	
	Other	0.8	1.7	1.2	
	Missing	0.5	0.3	0.4	
**DASS Depression Score (mean, CI)**		5.8	4.5	5.2	**Pr = 0.003**
**DASS Anxiety Score (mean, CI)**		3.3	2.7	3.0	**Pr = 0.012**
**DASS Stress Score (mean, CI)**		7.8	6.9	7.4	**Pr = 0.015**

Higher proportions of publicly insured and lower proportions of privately insured individuals lived in areas with less than 10% tree canopy. Race and ethnicity, educational attainment, and occupational status were not significantly different between the two types of environments. Continuous scores for depression, anxiety and stress differed significantly, with higher levels of each reported in areas with less than 10% tree canopy.

### 3.2. Models

Results indicated a largely consistent finding that higher levels of neighborhood green space are associated with better mental health outcomes, when considering a range of possible confounding factors. Results from analyses examining proportion tree canopy are presented in Table 2; full results from all models are included in Supplementary Materials. Findings were largely consistent across the three green space measures. Models are adjusted for all individual and neighborhood level characteristics displayed.

**Table 2:** Multivariate Linear Regression Models Linking Neighborhood Green Space to Mental Health **^§^**.

Variables		Depression	Anxiety	Stress
**Length of Residence**				
	Less than 1 Year in Current Residence	Referent	Referent	Referent
	1–2 Years	−1.573	−0.708	0.049
		(0.927)	(0.489)	(0.836)
	More than 2 but Fewer than 5 Years	−1.355	−0.601	−0.208
		(0.949)	(0.462)	(0.761)
	More than 5 but Fewer than 10 Years	−1.400	−0.676	−0.947
		(1.038)	(0.533)	(0.859)
	More than 10 but Fewer than 20 Years	−1.672	−0.978	−0.323
		(0.967)	(0.521)	(0.887)
	More than 20 but Fewer than 40 Years	−1.207	−0.696	−0.416
		(1.057)	(0.554)	(0.862)
	More than 40 Years	−1.704	−0.817	−0.971
		(0.953)	(0.442)	(0.786)
**Age **				
	Age 21 to 34	Referent	Referent	Referent
	Age 35 to 44	1.376	0.146	0.350
		(0.834)	(0.418)	(0.709)
	Age 45 to 54	1.076	−0.114	−1.185
		(0.612)	(0.393)	(0.556) *
	Age 55 to 64	−0.434	−0.444	−2.669
		(0.659)	(0.403)	(0.536) **
	Age 65 to 74	−5.308	−2.833	−6.166
		(1.063) **	(0.581) **	(0.805) **
**Sex**				
	Male	Referent	Referent	Referent
	Female	−0.852	−0.163	0.185
		(0.398) *	(0.262)	(0.398)
**Race and Ethnicity**				
	Non-Hispanic White	Referent	Referent	Referent
	Other Race/Ethnicity	−2.225	−0.409	−2.328
		(0.657) **	(0.419)	(0.638) **
**Education**				
	Less than High School Degree	0.069	1.174	0.433
		(0.671)	(0.636)	(0.693)
	High School Degree or GED	0.256	0.434	−0.067
		(0.594)	(0.327)	(0.496)
	Some College	Referent	Referent	Referent
	Bachelor’s Degree	−0.337	−0.155	0.063
		(0.450)	(0.250)	(0.513)
	Post-graduate Education	−0.791	−0.626	−0.436
		(0.424)	(0.254) *	(0.535)
**Income**				
	Less than $20,000	2.122	1.019	1.714
		(0.897) *	(0.499) *	(0.653) **
	$20,000–$34,999	1.193	0.797	0.583
		(0.769)	(0.496)	(0.714)
	$35,000–$49,999	0.910	0.445	0.519
		(0.600)	(0.382)	(0.617)
	$50,000–$74,999	0.726	0.389	0.648
		(0.568)	(0.295)	(0.464)
	$75,000 or more	Referent	Referent	Referent
**Marital Status**				
	Married	Referent	Referent	Referent
	Never married	−0.476	−0.281	−1.984
		(0.625)	(0.411)	(0.535) **
	Divorced, Separated or Widowed	1.221	0.677	−0.051
		(0.634)	(0.346)	(0.522)
**Employment Status**				
	Employed	Referent	Referent	Referent
	With Job/Business, but Not at Work	0.455	0.504	0.709
		(0.673)	(0.422)	(0.987)
	Unemployed and Looking for Work	2.357	0.675	1.321
		(1.322)	(0.720)	(1.184)
	Unemployed and Not Looking for Work	2.407	1.274	1.207
		(0.651) **	(0.365) **	(0.537) *
**Insurance Status**				
	Privately Insured	Referent	Referent	Referent
	Uninsured	2.054	0.487	1.486
		(0.956) *	(0.625)	(0.936)
	Publicly Insured	2.111	0.926	2.529
		(0.928) *	(0.508)	(0.915) **
	Multiple Sources of Insurance	3.976	1.974	2.792
		(1.192) **	(0.493) **	(0.675) **
	Other Insurance	3.957	3.151	4.493
		(2.133)	(1.650)	(1.728) *
**Urbanicity/Rurality**				
	Metropolitan	Referent	Referent	Referent
	Micropolitan	1.819	0.492	1.047
		(0.599) **	(0.351)	(0.480) *
	Small Town	0.664	0.546	0.065
		(0.517)	(0.396)	(0.630)
	Rural	2.773	0.719	1.014
		(1.008) **	(0.347) *	(0.548)
**Population Density**		−181.002	−140.529	−142.133
		(175.877)	(123.875)	(187.016)
Proportion Tract Population with High School Degree or Higher Education		−0.015	−0.032	−0.023
		(0.043)	(0.030)	(0.038)
Proportion Tract Population Living in Same House 1 Year Ago		−0.020	−0.017	−0.041
		(0.029)	(0.021)	(0.028)
Proportion Tract Population Unemployed		0.169	0.204	0.272
		(0.121)	(0.086) *	(0.105) *
Proportion Tract Population in Poverty		−0.067	−0.017	−0.037
		(0.042)	(0.029)	(0.041)
Proportion Tract Housing Owner-Occupied		−0.042	−0.012	0.006
		(0.019) *	(0.013)	(0.019)
Proportion Tract Population Black or African American		0.037	0.035	0.031
		(0.017) *	(0.014) *	(0.016)
Median Household Income (in $1000)		0.048	0.025	0.014
		(0.019) *	(0.014)	(0.019)
**Proportion Tree Canopy**		−4.020	−1.093	−2.193
		(1.172) **	(0.558)	(1.043) *
***R*^2^**		0.16	0.15	0.12
***N***		2,167	2,166	2,167

Notes: * *p* < 0.05; ** *p* < 0.01; **^§^** Numbers in the table represent the adjusted linear regression coefficient (in depression, anxiety, or stress score units) and its standard errors (in parenthesis) comparing each category to the referent category—adjusting for all the variables in the table.

#### 3.2.1. Individual Characteristics

As shown in Table 2, a number of socio-demographic factors are associated with mental health outcomes in Wisconsin. Coefficients can be interpreted as the difference in the mental health score given a one unit increase in the independent variable, meaning that negative coefficients represent better mental health, while positive coefficients indicate poorer mental health. Older age was associated with lower levels of depression, anxiety and stress. Gender was significant only for depression, with females being slightly but significantly less likely to report depressive symptomology. When compared to non-Hispanic whites, individuals of other racial and ethnic categories reported fewer symptoms of depression and stress, while there was no association between race/ethnicity and anxiety levels.

Educational attainment was only associated with anxiety, with evidence of a gradient effect, with individuals with less than a high school education reporting more anxiety and individuals with post-graduate education reporting less anxiety compared to a reference group of respondents who had some education post-high school but had not acquired a college degree. Income was related to all three mental health outcomes, with those making less than $20,000 per year reporting more symptoms than those making $75,000 or more per year.

Those who had never been married reported lower levels of stress; some evidence suggested that those who were divorced, separated or widowed experienced more symptoms of depression. Those uninsured, publicly insured, or with multiple sources of insurance reported more symptoms of depression than those privately insured. Only those with multiple sources of insurance reported significantly higher levels of anxiety. Those publicly insured, with multiple sources of insurance, or “other” sources reported higher levels of stress.

#### 3.2.2. Neighborhood Characteristics

Several neighborhood environmental factors were associated with poor mental health symptomology. Compared to a metropolitan reference group, individuals living in micropolitan settings reported higher levels of symptomology for depression and stress. In addition, those in rural areas reported higher levels of depression and anxiety. Higher neighborhood unemployment was associated with higher levels of anxiety and stress, while higher median household income was associated with higher levels of depression. Higher levels of residential racial segregation were also associated with depression, anxiety and stress. Higher proportions of owner occupied housing were associated with lower levels of depression. Population density, proportion with high school degree, residential instability, and poverty were not associated with any of the outcomes examined. Length of residence in a neighborhood was not significant in any of the models.

Neighborhood green space was consistently associated with lower levels of depression, anxiety and stress, when controlling for all individual and neighborhood characteristics, as displayed in Table 2. Depression showed the strongest relationship, with all three measures of greenspace significant, and depressive symptoms ranging from −4.02 to −5.515 lower for those in the greenest environment compared to those in the least green environment. Two of the three measures of green space were associated with significantly lower levels of stress and anxiety, with smaller differences in symptomology as compared to depression.

To enhance interpretability of our findings, we present coefficients illustrating the difference in symptom levels for a 25% change in greenspace in Table 3 (adjusting for the all the same variables in Table 2). A 25% increase in the proportion of tree canopy in a neighborhood is associated with a decrease in the DASS score for depression of approximately 1 point.

**Table 3:** Difference in Symptoms of Depression, Anxiety and Stress Associated with 25% More Neighborhood Green Space **^§§^**.

Green Space Measure	Depression	Anxiety	Stress
25% More Tree Canopy	−1.005 (0.293) **	−0.273 (0.139)	−0.548 (0.261) *
25% Higher NDVI	−1.369 (0.464) **	−0.512 (0.227) *	−0.701 (0.432)
25% More Greenspace (NDVI &Tree Canopy Average)	−1.379 (0.397) **	−0.427 (0.185) *	−0.735 (0.349) *

Notes: * *p* < 0.05; ** *p* < 0.01; **^§§^** Numbers in the table represent the adjusted linear regression coefficient (in depression, anxiety, or stress score units) and its standard errors (in parenthesis)—adjusting for all the variables displayed in Table 2.

## 4. Conclusions

We found in our sample that higher levels of neighborhood green space correspond to better mental health outcomes, when controlling for a wide range of confounding factors. The associations between green space and mental health are significant and sizeable and persist with different measurement techniques. Furthermore, the estimated effect of environmental green space is similar in magnitude to that of other well-known and studied contributors to symptomology for depression, anxiety and stress. For example, results indicate that the difference in depressive symptoms between an individual living in an environment with no tree canopy and an environment with 100% tree canopy is larger than the difference in symptoms associated with an individual who is uninsured compared to an individual with private insurance.

This study also revealed interesting differences in access to percentage of tree canopy based on demographic variations in population length of residence, age, income, marital status, and insurance type. Our multivariate adjusted results highlight that those from lower income brackets and without private health insurance experience greater anxiety, stress, and depression, thus supporting the notion that low socioeconomic populations could benefit more from increased exposure to green space [19]. This study found a higher percentage of tree canopy and more positive mental health among populations age 55 and older. In other literature, older populations have been identified along with individuals of low socioeconomic status as being vulnerable and likely to reap increased benefits from green space based on increased time spent in their neighborhoods [19]. In this study, it appears that younger adults may currently experience greater need to receive the mental health benefits conferred by greener environments. Future research should continue to explore how demographic characteristics such as race, age, and income contribute to both access to green space and mental health benefits of green spaces.

Another important contribution of this study is the examination of neighborhood environmental factors other than green space. The correlation between higher unemployment levels and greater anxiety and stress and the correlation between higher levels of residential segregation and increased anxiety, stress, and depression support the need for research on “greening” and other neighborhood strategies to counteract spatial aspects of socioeconomic disadvantage [7,16,19,56,63]. Our finding of higher levels of depression among rural populations highlights potential benefits of environmental green spaces for diverse populations and motivates the expansion of green space research to non-urban environments, as well as the examination of benefits by socio-demographic and economic characteristics of residents.

Strengths of this study include the population-based sample, the use of state-of-the art and detailed measures of green space, the use of well-known and validated outcome measures (DASS instruments), and the consideration of a large number of covariates to control for confounding influences. Several potential limitations of this study are worth considering. SHOW data are based on self-reported health outcomes and non-response might introduce selection bias. The use of SHOW data from 2008–2011 with satellite data from 2009 and tree canopy data from 2001 might introduce some error stemming from temporal misalignment between survey responses and neighborhood greenness. We limited NDVI analyses to 2009 due to the availability of images that were not significantly affected by cloud cover. As NDVI calculations for 2009 and percent tree canopy for 2001 are highly correlated (*r* = 0.83), we assume that temporal misalignment does not alter the results of the study significantly. Similarly, the unavailability of Census Block Group information and resultant use of Census Tract data for estimating neighborhood socioeconomic characteristics may introduce some error stemming from spatial misalignment of predictors and outcomes.

A final and important limitation is the problem of reverse causality or neighborhood selection bias, whereby it is possible that associations found could in part be attributable to populations with fewer mental health problems moving into greener environments, given that this is a cross-sectional study [64]. While this possibility cannot be ruled out, the body of evidence, including experimental work, linking exposure to green space and nature to mental health benefits provides strong support for a causal relationship between greenspace and mental health, which should be further investigated. In addition, we controlled for length of residence in the neighborhood to reduce the impact of neighborhood selection bias.

Previous work has identified “a positive relation between perceived general health and both agricultural green and natural green in a person’s living environment” [19]. Building upon this previous work, we contribute, to our knowledge, the first investigation of the relationships between green space and mental health in a study area that includes urban and rural landscapes common across the United States. Our work indicates that “greening” could be considered a potentially low cost, high return investment among urban and regional planners to positively influence population mental health. Further, it is know that mental health conditions such as stress, anxiety and depression can be associated with a myriad of other adverse health conditions, missed days of work and low productivity, indicating the benefits of such a strategy could be diverse and numerous. More research in this area is needed, particularly as some have suggested the potentially negative aspects of greener environments, including their association with sprawl and possibly diseases associated with sedentary behaviors encouraged by car dependent environments [48].

Given an evidence base suggesting associations between environmental green space and both physical and mental health outcomes in non-US settings, this work provides further evidence to support neighborhood level greening as a potentially important intervention for improving health and reducing persistent disparities. Furthermore, because of significant and persistent patterns of place-based health inequities associated with the history of residential racial segregation in the US, which often coincide with adverse social and build environments, there is a great need for continued research examining greening as a population health intervention in the US.

## Supplementary Files

Supplementary File 1 Supplementary Information (PDF, 160 KB)
