# Supplementary material for: Exposure to Neighborhood Green Space and Mental Health: Evidence from the Survey of the Health of Wisconsin
Source: Int J Environ Res Public Health. 2014 Mar 21;11(3):3453–72. doi: 10.3390/ijerph110303453 (PMC3987044; doi:10.3390/ijerph110303453)
Supplement: Supplementary File 1 — Supplementary Information (PDF, 160 KB) [file ijerph-11-03453-s001.pdf]

Exposure to Neighborhood Green Space and Mental Health: Evidence from the Survey of the Health of Wisconsin

Table S1. Multivariate Linear Regression—Models Linking Neighborhood Green Space to Mental Health §.

Variables	Depression			Anxiety			Stress		
Less than 1 Year in Current Residence	Referent			Referent			Referent		
1−2 Years	−1.573	−1.557	−1.555	−0.708	−0.697	−0.700	0.049	0.056	0.058
	(0.927)	(0.913)	(0.916)	(0.489)	(0.487)	(0.488)	(0.836)	(0.832)	(0.832)
More than 2 but Fewer than 5 Years	−1.355	−1.364	−1.380	−0.601	−0.615	−0.613	−0.208	−0.210	−0.220
	(0.949)	(0.955)	(0.954)	(0.462)	(0.461)	(0.463)	(0.761)	(0.762)	(0.762)
More than 5 but Fewer than 10 Years	−1.400	−1.438	−1.421	−0.676	−0.692	−0.683	−0.947	−0.966	−0.958
	(1.038)	(1.040)	(1.034)	(0.533)	(0.531)	(0.532)	(0.859)	(0.859)	(0.858)
More than 10 but Fewer than 20 Years	−1.672	−1.683	−1.667	−0.978	−0.976	−0.974	−0.323	−0.330	−0.321
	(0.967)	(0.957)	(0.957)	(0.521)	(0.517)	(0.519)	(0.887)	(0.884)	(0.883)
More than 20 but Fewer than 40 Years	−1.207	−1.237	−1.228	−0.696	−0.710	−0.703	−0.416	−0.431	−0.427
	(1.057)	(1.058)	(1.056)	(0.554)	(0.554)	(0.554)	(0.862)	(0.864)	(0.862)
More than 40 Years	−1.704	−1.754	−1.718	−0.817	−0.829	−0.819	−0.971	−0.998	−0.979
	(0.953)	(0.948)	(0.946)	(0.442)	(0.440)	(0.441)	(0.786)	(0.784)	(0.784)
Age 21 to 34	Referent			Referent			Referent		
Age 35 to 44	1.376	1.397	1.375	0.146	0.148	0.144	0.350	0.363	0.351
	(0.834)	(0.830)	(0.830)	(0.418)	(0.419)	(0.418)	(0.709)	(0.711)	(0.708)
Age 45 to 54	1.076	1.064	1.080	−0.114	−0.113	−0.111	−1.185	−1.193	−1.184
	(0.612)	(0.605)	(0.609)	(0.393)	(0.392)	(0.393)	(0.556) *	(0.556) *	(0.556) *
Age 55 to 64	−0.434	−0.470	−0.434	−0.444	−0.448	−0.440	−2.669	−2.691	−2.670
	(0.659)	(0.657)	(0.660)	(0.403)	(0.404)	(0.404)	(0.536) **	(0.537) **	(0.538) **
Age 65 to 74	−5.308	−5.358	−5.315	−2.833	−2.842	−2.831	−6.166	−6.195	−6.171
	(1.063) **	(1.064) **	(1.059) **	(0.581) **	(0.581) **	(0.581) **	(0.805) **	(0.806) **	(0.807) **

Table S1. *Cont.*

**Variables**	**Depression**			**Anxiety**			**Stress**		
Male	Referent			Referent			Referent		
Female	−0.852	−0.838	−0.847	−0.163	−0.159	−0.162	0.185	0.192	0.187
	(0.398) *	(0.401) *	(0.400) *	(0.262)	(0.264)	(0.263)	(0.398)	(0.399)	(0.399)
Non−Hispanic White	Referent			Referent			Referent		
Other Race/Ethnicity	−2.225	−2.273	−2.234	−0.409	−0.419	−0.409	−2.328	−2.355	−2.334
	(0.657) **	(0.661) **	(0.656) **	(0.419)	(0.422)	(0.420)	(0.638) **	(0.641) **	(0.636) **
Less than High School Degree	0.069	−0.010	0.025	1.174	1.141	1.159	0.433	0.394	0.410
	(0.671)	(0.676)	(0.672)	(0.636)	(0.635)	(0.635)	(0.693)	(0.690)	(0.691)
High School Degree or GED	0.256	0.210	0.235	0.434	0.418	0.428	−0.067	−0.091	−0.079
	(0.594)	(0.597)	(0.594)	(0.327)	(0.327)	(0.327)	(0.496)	(0.498)	(0.496)
Some College	Referent			Referent			Referent		
Bachelor's Degree	−0.337	−0.368	−0.354	−0.155	−0.167	−0.160	0.063	0.048	0.054
	(0.450)	(0.455)	(0.451)	(0.250)	(0.251)	(0.250)	(0.513)	(0.518)	(0.514)
Post−graduate Education	−0.791	−0.791	−0.770	−0.626	−0.615	−0.615	−0.436	−0.440	−0.427
	(0.424)	(0.416)	(0.418)	(0.254) *	(0.255) *	(0.255) *	(0.535)	(0.530)	(0.533)
Income less than $20,000	2.122	2.119	2.116	1.019	1.016	1.017	1.714	1.713	1.711
	(0.897) *	(0.891) *	(0.895) *	(0.499) *	(0.497) *	(0.498) *	(0.653) **	(0.651) **	(0.651) **
$20,000−$34,999	1.193	1.185	1.196	0.797	0.798	0.800	0.583	0.578	0.584
	(0.769)	(0.768)	(0.769)	(0.496)	(0.494)	(0.496)	(0.714)	(0.714)	(0.714)
$35,000−$49,999	0.910	0.925	0.928	0.445	0.457	0.453	0.519	0.525	0.529
	(0.600)	(0.609)	(0.605)	(0.382)	(0.383)	(0.382)	(0.617)	(0.615)	(0.617)
$50,000−$74,999	0.726	0.734	0.735	0.389	0.394	0.392	0.648	0.652	0.653
	(0.568)	(0.570)	(0.568)	(0.295)	(0.295)	(0.295)	(0.464)	(0.466)	(0.465)
$75,000 or more	Referent			Referent			Referent		

Table S1. *Cont.*

**Variables**	**Depression**			**Anxiety**			**Stress**		
Married	Referent			Referent			Referent		
Never married	−0.476	−0.457	−0.487	−0.281	−0.285	−0.288	−1.984	−1.970	−1.988
	(0.625)	(0.618)	(0.624)	(0.411)	(0.412)	(0.412)	(0.535) **	(0.537) **	(0.537) **
Divorced, Separated or Widowed	1.221	1.254	1.221	0.677	0.680	0.674	−0.051	−0.032	−0.050
	(0.634)	(0.633) *	(0.636)	(0.346)	(0.346)	(0.346)	(0.522)	(0.525)	(0.523)
Employed	Referent			Referent			Referent		
With Job/Business, but Not at Work	0.455	0.412	0.427	0.504	0.485	0.494	0.709	0.688	0.695
	(0.673)	(0.638)	(0.658)	(0.422)	(0.415)	(0.419)	(0.987)	(0.975)	(0.981)
Unemployed and Looking for Work	2.357	2.329	2.342	0.675	0.664	0.670	1.321	1.307	1.313
	(1.322)	(1.324)	(1.323)	(0.720)	(0.724)	(0.721)	(1.184)	(1.188)	(1.185)
Unemployed and Not Looking for Work	2.407	2.435	2.414	1.274	1.281	1.274	1.207	1.223	1.211
	(0.651) **	(0.649) **	(0.649) **	(0.365) **	(0.363) **	(0.364) **	(0.537) *	(0.537) *	(0.536) *
Privately Insured	Referent			Referent			Referent		
Uninsured	2.054	1.968	2.036	0.487	0.469	0.487	1.486	1.438	1.475
	(0.956) *	(0.956) *	(0.956) *	(0.625)	(0.624)	(0.625)	(0.936)	(0.938)	(0.937)
Publicly Insured	2.111	2.069	2.093	0.926	0.912	0.921	2.529	2.507	2.520
	(0.928) *	(0.935) *	(0.932) *	(0.508)	(0.511)	(0.509)	(0.915) **	(0.916) **	(0.917) **
Multiple Sources of Insurance	3.976	3.929	3.947	1.974	1.953	1.964	2.792	2.769	2.777
	(1.192) **	(1.182) **	(1.182) **	(0.493) **	(0.489) **	(0.491) **	(0.675) **	(0.669) **	(0.671) **
Other Insurance	3.957	3.735	3.868	3.151	3.079	3.128	4.493	4.375	4.444
	(2.133)	(2.167)	(2.141)	(1.650)	(1.664)	(1.652)	(1.728) *	(1.744) *	(1.734) *
Metropolitan	Referent			Referent			Referent		
Micropolitan	1.819	1.865	1.860	0.492	0.518	0.508	1.047	1.067	1.067
	(0.599) **	(0.573) **	(0.584) **	(0.351)	(0.346)	(0.349)	(0.480) *	(0.463) *	(0.471) *
Small Town	0.664	0.758	0.745	0.546	0.600	0.577	0.065	0.107	0.106
	(0.517)	(0.513)	(0.515)	(0.396)	(0.403)	(0.400)	(0.630)	(0.631)	(0.634)

Table S1. *Cont.*

**Variables**	**Depression**			**Anxiety**			**Stress**		
Rural	2.773	2.820	2.866	0.719	0.774	0.762	1.014	1.026	1.059
	(1.008) **	(1.019) **	(0.995) **	(0.347) *	(0.340) *	(0.342) *	(0.548)	(0.551)	(0.542)
Population Density	−181.002	−205.624	−203.066	−140.529	−154.902	−149.267	−142.133	−153.016	−153.273
	(175.877)	(179.829)	(176.809)	(123.875)	(123.878)	(123.571)	(187.016)	(190.198)	(187.693)
Proportion Tract Population with High School Degree or Higher Education	−0.015	−0.019	−0.013	−0.032	−0.031	−0.031	−0.023	−0.025	−0.022
	(0.043)	(0.042)	(0.042)	(0.030)	(0.031)	(0.030)	(0.038)	(0.038)	(0.038)
Proportion Tract Population Living in Same House 1 Year Ago	−0.020	−0.026	−0.024	−0.017	−0.020	−0.018	−0.041	−0.043	−0.043
	(0.029)	(0.030)	(0.029)	(0.021)	(0.021)	(0.021)	(0.028)	(0.029)	(0.028)
Proportion Tract Population Unemployed	0.169	0.173	0.175	0.204	0.208	0.207	0.272	0.273	0.275
	(0.121)	(0.119)	(0.119)	(0.086) *	(0.085) *	(0.085) *	(0.105) *	(0.105) *	(0.105) **
Proportion Tract Population in Poverty	−0.067	−0.070	−0.065	−0.017	−0.017	−0.016	−0.037	−0.040	−0.036
	(0.042)	(0.040)	(0.041)	(0.029)	(0.030)	(0.029)	(0.041)	(0.041)	(0.041)
Proportion Tract Housing Owner−Occupied	−0.042	−0.040	−0.038	−0.012	−0.010	−0.010	0.006	0.006	0.008
	(0.019) *	(0.019) *	(0.018) *	(0.013)	(0.013)	(0.013)	(0.019)	(0.019)	(0.019)
Proportion Tract Population Black or African American	0.037	0.040	0.037	0.035	0.036	0.035	0.031	0.033	0.032
	(0.017) *	(0.016) *	(0.016) *	(0.014) *	(0.014) **	(0.014) *	(0.016)	(0.016) *	(0.016)
Median Household Income (in $1000)	0.048	0.052	0.049	0.025	0.026	0.025	0.014	0.017	0.015
	(0.019) *	(0.019) **	(0.019) **	(0.014)	(0.013)	(0.014)	(0.019)	(0.019)	(0.019)
Proportion Tree Canopy	−4.020			−1.093			−2.193		
	(1.172) **			(0.558)			(1.043) *		
NDVI: No Clouds, No Water		−5.476			−2.049			−2.802	
		(1.854) **			(0.910) *			(1.729)	
Average: NDVI and Tree Canopy			−5.515			−1.706			−2.941
			(1.590) **			(0.740) *			(1.397) *

Table S1. *Cont.*

Variables	Depression			Anxiety			Stress		
R^2	0.16	0.16	0.16	0.15	0.15	0.15	0.12	0.12	0.12
N	2,167	2,167	2,167	2,166	2,166	2,166	2,167	2,167	2,167

Notes: * $p < 0.05$; ** $p < 0.01$; [§] Numbers in the table represent the adjusted linear regression coefficient (in depression, anxiety, or stress score units) and its standard errors (in parenthesis) comparing each category to the referent category—adjusting for all the variables in the table.

© 2014 by the authors; licensee MDPI, Basel, Switzerland. This article is an open access article distributed under the terms and conditions of the Creative Commons Attribution license (http://creativecommons.org/licenses/by/3.0/).
